# Green ecofriendly enhancement of cellulase productivity using agricultural wastes by *Aspergillus terreus* MN901491: statistical designs and detergent ability on cotton fabrics

**DOI:** 10.1186/s12934-024-02376-3

**Published:** 2024-04-12

**Authors:** Mohamed A.A. Abdella, Nehad E. Ahmed, Mohamed S. Hasanin

**Affiliations:** 1 Chemistry of Natural and Microbial Products Department, Pharmaceutical and Drug Industries Research Institute, National Research Centre, Dokki, Giza, 12622 Egypt; 2 Cellulose and Paper Department, National Research Centre, Dokki, Giza, 12622 Egypt

**Keywords:** Cellulase, Agricultural wastes, Statistical designs, Optimization, Bio-additive detergent

## Abstract

**Background:**

Cellulase is considered a group member of the hydrolytic enzymes, responsible for catalyzing the hydrolysis of cellulose and has various industrial applications. Agricultural wastes are used as an inexpensive source for several utilizable products throughout the world. So, searching for cellulase enzymes from fungal strains capable of utilizing agricultural wastes to increase productivity, reduce costs and overcome waste accumulation in the environment is very important to evaluate its potency as a bio-additive to detergent agents.

**Results:**

In the current study, the previously identified fungal strain *Aspergillus terreus* MN901491 was screened and selected for cellulase production. Medium parameters were optimized using one-factor-at-a-time (OFAT) and multi-factorial (Plackett-Burman and Box-Behnken) design methods. OFAT showed the ability of the fungal strain to utilize agricultural wastes (corn cob and rice straw) as a substrate. Also, yeast extract was the best nitrogen source for enhancing cellulase productivity. The most significant variables were determined by Plackett-Burman Design (PBD) and their concentrations were optimized by Response Surface Methodology (RSM) using Box-Behnken Design (BBD). Among eleven independent variables screened by PBD, malt extract, (NH_4_)_2_SO_4_, and KCl were the most significant ones followed by rice straw which affected cellulase production positively. The ANOVA results particularly the R^2^-value of PBD (0.9879) and BBD (0.9883) confirmed the model efficiency and provided a good interpretation of the experiments. PBD and BBD improved cellulase productivity by 6.1-fold greater than that obtained from OFAT. Medium optimization using OFAT and statistical models increased cellulase production from *A. terreus* MN901491 by 9.3-fold compared to the non-optimized medium. Moreover, the efficiency of cellulase activity on cotton fabrics as a bio-additive detergent was evaluated and estimated using whiteness and scanning electron microscope (SEM) that affirmed its potential effect and remarkable detergent ability to improve whiteness by 200% in comparison with non-washed fabric and by 190% in comparison with fabric washed by water.

**Conclusion:**

The presented work was stabilized as a multi-efficiency in which wastes were used to produce cellulase enzyme from the fungal strain, *Aspergillus terreus* MN901491 as a bio-additive to detergent applications that involved ecofriendly and green processes.

## Introduction

Agricultural wastes including lignocellulosic materials are utilized as cheap resources for obtaining various beneficial products. Huge amounts of lignocellulosic wastes are the outcome of agricultural practices, forestry, and some industrial processes [1, 2]. Cellulose, hemicellulose and lignin are considered the main components of lignocellulosic materials and represent the most plenteous renewable carbon source in nature. Cellulose is a carbohydrate complex (polysaccharide), that has a linear series of numerous glucose units, linked together by β-(1,4)-glucosidic bonds [3, 4]. Many governments around the world are doing their best to get rid of environmental pollutants and looking for modern strategies to convert lignocellulosic biomass into useful products [2]. In lignocellulosic materials, polysaccharides could be hydrolyzed into simple sugars (monosaccharides) that could be utilized for different commodities output including ethanol, butanol, lactic acid, fatty acids, ethyl esters, and hydrogen gas [5–7]. Enzymes can subrogate the use of traditional hazardous chemical catalysts in various industrial fields which in turn make any operation more ecofriendly and economically feasible [8]. The complete hydrolysis of lignocellulosic polysaccharides is accomplished by a mix of hydrolyzing enzymes called cellulases and hemicellulases [9, 10].

Cellulases are divided into three major hydrolytic enzymes: (1) Endoglucanase or CMCase (*EC 3.2.1.4*) can randomly cleave cellulose series at the interior β-(1,4)-glucosidic linkages to give new ends including glucose units (2) Exoglucanase (*EC 3.2.1.91*) can hydrolyze both reducing and non-reducing terminals in cellulose polymer releasing end product of cellobiose (3) β-glucosidase (*EC 3.2.1.21*) breaks the cellobiose residues producing glucose [11, 12]. The importance of cellulases lies in various industrial applications like paper, textiles, food, beverages, detergents, nutrition, pharmaceuticals, and biofuel (alternate energy) production [4, 13]. They can result from a set of living organisms such as animals, plants, protozoa, fungi, and bacteria. However, microbial cellulases are superior to plant and animal ones due to the ease of their handling, fast growth, the ease of genetic material manipulation, great enzymatic stability under harsh conditions, simple and low production cost [14]. Further, cellulases obtained from fungi are preferable as compared to other microorganisms because of the higher rate of enzyme production. Many fungal genera comprising *Trichoderma*, *Aspergillus*, *Penicillium* and *Humicola* can produce cellulases, but only a few of them are capable of producing the enzyme in a considerable amount and can degrade crystalline cellulose [4, 12, 15].

The optimization of culture components and conditional parameters has a substantial turn for improving enzyme yield and reducing production costs [16]. The traditional one-factor-at-a-time (OFAT) method for medium optimization only is expensive, time intensive, exhausting, and disadvantageous to investigate a large number of variables [17]. Therefore, an appropriate strategy is required to design the optimization process that affects the final yield of the enzyme [4]. Multi-factorial (statistical) models [Plackett-Burman design (PBD) and Box-Behnken design (BBD) of Response Surface Methodology (RSM)] are successful tools for testing a great number of different variables, determining the most significant ones and defining their ideal levels to increase enzyme productivity. Furthermore, the statistical methods aid in decreasing the number of trials needed for examining the association between different variables that influence enzyme synthesis [3]. Herein, the detergent ability of some enzymes was detected and applied in many industrial applications [18]. In this context, the ability of cellulase enzyme to act as a detergent mainly depended on the cutting efficiency of the cellulose fibers’ tiny terminals that are attached to undesirable particles [19]. In summary, biological detergents have enzymes in them that facilitate the breakdown of filth that accumulates in your clothing. Because they don’t include synthetic chemicals are typically gentler on delicate skin [20]. Thus, the yield of cellulase enzyme by a fungal strain able to utilize agricultural wastes for increasing productivity, lowering the cost, and solving the waste accumulation problem is so significant to assess its potential as a bio-additive to the detergent agents.

This work aimed to optimize cellulase productivity from previously identified fungal strain *Aspergillus terreus* MN901491 initially by using the OFAT method to select the preferable sources of carbon and nitrogen for enzymatic synthesis. Statistical design (PBD and BBD) methods were implemented to enhance cellulase productivity. First, PBD was carried out to identify the significant variables followed by BBD for further optimization of the selected variables to maximize enzyme yield. Finally, the detergent ability of cellulase activity on cotton fabrics was estimated using whiteness and SEM techniques.

## Materials and methods

### Microorganism

The fungal strain, *Aspergillus terreus* MN901491 which was isolated and identified as described previously [21], was used for enzyme production. The current study protocol has an approval number of (27,447,082,023) from the Ethics Committee of the National Research Centre, Cairo, Egypt. The fungus was grown on PDA (Potato dextrose agar) plates at 30 °C for 7 days and then maintained at 4 °C.

### Demonstration of cellulase (CMCase) activity

The fungal strain (*A. terreus* MN901491) was investigated for cellulase production based on the method of Farkas et al. [22] using Congo red (CR) staining solution. Modified Czapek-Dox agar medium has carboxymethyl cellulose (CMC) as a carbon source was utilized, and comprised the following components (g/L): CMC, 10; NaNO_3_, 2; MgSO_4_.7H_2_O, 0.5; K_2_HPO_4_, 1; FeSO_4_, 0.01; KCl, 0.5; and agar 20, at pH 6. After sterilization, the medium was poured into plates, and the fungal strain was cultivated on the solidified medium as spore suspensions of the pure culture then, incubated for 5 days at 30 °C. The qualitative screening was done by flooding the plates with 1% CR solution (10 mL /15 min) and they were de-stained by the addition of NaOH solution 1 N (15 mL /30 min). Finally, the cellulolytic activity was checked through the formation of a halo zone around the fungus colonies [23].

### Cellulase production by the fungal strain

The positive cellulolytic fungal strain *A. terreus* MN901491 was cultivated in the basal medium under submerged fermentation (SmF). Cultivation was done with 2.0 mL spore suspension (2 × 10^6^ spore/mL) in modified Czapek-Dox broth medium [24] which composed of (g/L): CMC, 10; NaNO_3_, 2; MgSO_4_.7H_2_O, 0.5; K_2_HPO_4_, 1; FeSO_4_, 0.01; KCl, 0.5 (pH 6) and the flasks were put in a rotary shaker (150 rpm) at 30 °C for 5 days. After the fermentation period, the medium underwent centrifugation at 8,000 xg for 10 min under 4 °C and the obtained supernatant (crude cellulase extract) was introduced for further examinations.

### Cellulase (CMCase) activity estimation

Cellulolytic activity assay was done as reported by Zhang et al. [25] with some modifications. The reaction mixture contains 1 mL cellulase extract in addition to 1 mL CMC 1% (w/v) solution (dissolved in 50 mM sodium acetate buffer, pH 5). After that, the mix was left in a water bath for 30 min at 50 °C and the reduced sugar was assayed via 3,5 dinitrosalicylic acid (DNS) procedure as described by Miller [26]. Finally, cellulase activity was determined by measuring color absorbance at 540 nm using a spectrophotometer. The amount of enzyme that releases one (1) µmole of glucose per min under assessment conditions was known as one unit of enzyme activity.

### One- factor- at a time (OFAT) for optimizing cellulase productivity

#### Influence of various carbon sources on cellulase productivity

Several agricultural wastes such as (wheat bran, rice straw, rice bran, corn cob, potato peel, orange peel) and other compounds like (lactose, fructose, glucose, CMC) were separately added and used as different carbon sources [17] for cellulase production. The agriculture wastes were locally obtained and before use, they were washed, dried in an oven at 60 °C and grinded by blender. The CMC in the basal medium was substituted with an equal amount (1%) of other carbon sources. The activity of cellulase was assayed after incubating the inoculated medium at 30 °C in a rotary shaker (150 rpm) for 5 days.

#### Influence of various nitrogen sources on cellulase production

The fermentative medium was supplemented with organic (beef extract, malt extract, yeast extract, tryptone, peptone) and inorganic (KNO_3_, (NH_4_)_2_SO_4_, urea, NaNO_3_) compounds as different nitrogen sources [24]. Similarly, sodium nitrate (NaNO_3_) was replaced with an equal amount (0.2%) of other nitrogen sources and cellulase activity was determined after incubation at 30 °C for 5 days under 150 rpm. Finally, the best sources of carbon and nitrogen were further chosen to get the highest cellulase productivity.

### Statistical designs for cellulase production optimization

#### Plackett-Burman design (PBD)

PBD is a dominant way to select the significant parameters affecting cellulase productivity by *A. terreus* MN901491 [27]. In this design, 11 variables involving: corn cob, rice straw, yeast extract, malt extract, K_2_HPO_4_, MgSO_4_.7H_2_O, KCl, (NH_4_)_2_SO_4_, CaCl_2_, inoculum size and incubation time were evaluated. These variables were chosen based on the basal medium components, the best carbon and nitrogen sources of OFAT optimization, and the literature review. The ranges of all variables were examined at both low (−) and high (+) levels (Table 1). The number of total runs in PBD was established by the equation: R = *n* + 1 where, R (runs number) and n (variables number). Also, in the PBD, the experiments were represented by rows, while independent variables were represented by columns and cellulase activity (U/mL) was used as a response (Table 2). The next first-order equation was exercised to explain the PBD model:

1 $${\rm{Y}}\,{\rm{ = }}\,{{\rm{\beta }}_{\rm{0}}}\,{\rm{ + }}\,\sum {{{\rm{\beta }}_{\rm{i}}}\,{{\rm{X}}_{\rm{i}}}}$$

Where: Y (the response), β_0_ (the intercept of the model), β_i_ (linear coefficient) and X_i_ (the level of variables). Also, the analysis of variance (ANOVA) of PBD was displayed in Table 3.

**Table 1:** PBD showing different variables and their tested levels

Variable		Unit	Level	
Name	Code		Low [−]	High [+]
Corn cob	A	%	0.5	1.5
Rice straw	B	%	0	0.5
Yeast extract	C	%	0.2	0.5
Malt extract	D	%	0	0.5
K_2_HPO_4_	E	%	0.05	0.2
MgSO_4_.7H_2_O	F	%	0.025	0.1
KCl	G	%	0.025	0.075
(NH_4_)_2_SO_4_	H	%	0	0.1
CaCl_2_	J	%	0	0.1
Incubation time	K	day	5	8
Inoculum size	L	%	2	4

**Table 2:** PBD for screening 11 variables affecting cellulase production by *A. terreus* MN901491

Run	A	B	C	D	E	F	G	H	J	K	L	Cellulase activity	Predicted value
	%	%	%	%	%	%	%	%	%	day	%	U/mL	U/mL
1	0.5	0	0.2	0.5	0.05	0.1	0.075	0	0.1	8	4	141.4	143.2
2	1.5	0.5	0.2	0	0.05	0.1	0.025	0.1	0.1	5	4	54	55.8
3	0.5	0.5	0.2	0.5	0.2	0.025	0.075	0.1	0.1	5	2	264.9	266.7
4	0.5	0	0.2	0	0.05	0.025	0.025	0	0	5	2	38.3	33.4
5	0.5	0	0.5	0	0.2	0.1	0.025	0.1	0.1	8	2	15.4	20.3
6	1.5	0.5	0.5	0	0.05	0.025	0.075	0	0.1	8	2	53.2	58.1
7	1.5	0	0.5	0.5	0.2	0.025	0.025	0	0.1	5	4	42.2	47.1
8	1.5	0	0.2	0	0.2	0.025	0.075	0.1	0	8	4	47.8	42.9
9	1.5	0	0.5	0.5	0.05	0.1	0.075	0.1	0	5	2	212.6	210.8
10	1.5	0.5	0.2	0.5	0.2	0.1	0.025	0	0	8	2	54.2	49.3
11	0.5	0.5	0.5	0.5	0.05	0.025	0.025	0.1	0	8	4	200	198.2
12	0.5	0.5	0.5	0	0.2	0.1	0.075	0	0	5	4	58.6	56.78

**Table 3:** ANOVA of PBD for cellulase production by *A. terreus* MN901491

Source	Sum of Squares	DF	Mean Square	Std. Dev.	*F*-value	*P*-value	
Model	76542.23	9	8504.69	9.08	103.19	0.0096	Significant
A-Corn cob	5401.76	1	5401.76	0.5222	65.54	0.0149	
B-Rice straw	2920.32	1	2920.32	0.2611	35.43	0.0271	
D-Malt extract	34992.00	1	34992.00	0.2611	424.55	0.0023	
E-K_2_HPO_4_	3902.41	1	3902.41	0.0783	47.35	0.0205	
F-MgSO_4_.7HO	1012.00	1	1012.00	0.0392	12.28	0.0727	
G-KCl	11681.28	1	11681.28	0.0261	141.73	0.0070	
H-(NH_4_)_2_SO_4_	13790.52	1	13790.52	0.0522	167.32	0.0059	
K-Incubation time	2096.16	1	2096.16	1.57	25.43	0.0371	
L-Inoculum size	745.76	1	745.76	1.04	9.05	0.0950	
Residual	164.84	2	82.42				
Cor Total	76707.07	11					

*R²***=** 0.9879, Adjusted *R*^*2*^ = 0.9782, Predicted *R*^*2*^ = 0.9226, CV = 9.21%, Std. Dev. (standard deviation),

DF (degree of freedom), Significant (*P* < 0.05), Non-significant (*P* > 0.05)

#### Box-Behnken Design (BBD)

BBD of Response Surface Methodology (RSM) proceeded after the first-order screening PBD to get the best concentrations of significant variables for increasing enzyme yield [28]. Based on PBD results, 3 variables (malt extract, (NH_4_)_2_SO_4_, and KCl) were found to be the most effective factors that were further examined at low (-), central (0), and high (+) levels (Table 3). By constructing BBD, fifteen runs were generated as a result of combinations between variable levels concerning response that was expressed by the mean of cellulase activity (U/mL) as shown in Table 4. The BBD data were demonstrated by the following second-order model:

2 $${\rm{Y}}\,{\rm{ = }}\,{{\rm{\beta }}_{\rm{0}}}\,{\rm{ + }}\,\sum {{{\rm{\beta }}_{\rm{i}}}{{\rm{X}}_{\rm{i}}}} \,{\rm{ + }}\,\sum {{{\rm{\beta }}_{{\rm{ii}}}}{\rm{X}}_{\rm{i}}^2} \,{\rm{ + }}\,\sum {{{\rm{\beta }}_{{\rm{ij}}}}{{\rm{X}}_{\rm{i}}}{{\rm{X}}_{\rm{j}}}}$$

Where: Y (the response), β_0_ (the intercept of the model), β_i_ (the linear coefficient), β_ii_ (the squared coefficient), β_ij_ (the interaction coefficient), X_i_, X_j_ (the independent variables). Additionally, Table 6 offers the ANOVA of the BBD model. For experimental design and statistical interpretations, the Design Expert program (version 13.0, Stat Ease Inc., Minneapolis, MN, USA) was applied.

**Table 4:** BBD for the selected variables and their different levels

Variable	Code	Unit	Level		
			Low [−]	Central [0]	High [+]
Malt extract	A	%	0.5	1	1.5
(NH_4_)_2_SO_4_	B	%	0.1	0.3	0.5
KCl	C	%	0.1	0.25	0.4

**Table 5:** BBD for optimizing variables affecting cellulase productivity by *A. terreus* MN901491

Run	A: Malt extract	B:(NH_4_)_2_SO_4_	C: KCl	Cellulase activity	Predicted values
	%	%	%	U/mL	U/mL
1	0.5	0.3	0.4	156.4	170.33
2	1	0.3	0.25	412.9	405.67
3	0.5	0.5	0.25	345.3	330.98
4	1	0.3	0.25	405.9	405.67
5	1	0.1	0.4	369.5	366.00
6	1	0.5	0.1	410.2	413.70
7	1.5	0.1	0.25	442.1	456.43
8	1.5	0.3	0.1	357.4	343.48
9	0.5	0.3	0.1	175.6	186.43
10	1	0.1	0.1	384	383.60
11	1.5	0.5	0.25	343	353.43
12	0.5	0.1	0.25	187.4	176.98
13	1	0.5	0.4	386.5	386.90
14	1	0.3	0.25	398.2	405.67
15	1.5	0.3	0.4	326	315.17

**Table 6:** ANOVA of BBD for optimizing cellulase productivity by *A. terreus* MN901491

Source	Sum of Squares	DF	Mean Square	Std. Dev.	*F*-value	*P*-value	
Model	1.165E + 05	9	12949.05	16.63	46.82	0.0003	Significant
A- Malt extract	45571.80	1	45571.80	0.3780	164.77	< 0.0001	
B-(NH_4_)_2_SO_4_	1300.50	1	1300.50	0.1512	4.70	0.0823	
C-KCl	985.68	1	985.68	0.1134	3.56	0.1177	
AB	16512.25	1	16512.25		59.70	0.0006	
AC	37.21	1	37.21		0.1345	0.7288	
BC	21.16	1	21.16		0.0765	0.7932	
A²	40675.39	1	40675.39		147.07	< 0.0001	
B²	3050.15	1	3050.15		11.03	0.0210	
C²	8107.21	1	8107.21		29.31	0.0029	
Residual	1382.89	5	276.58				
Lack of Fit	1274.77	3	424.92		7.86	0.1150	Non-significant
Pure Error	108.13	2	54.06				
Cor Total	1.179E + 05	14					

*R²***=** 0.9883, Adjusted *R*^*2*^ = 0.9672, Predicted *R*^*2*^ = 0.8250, CV = 4.89%, Std. Dev. (standard deviation),

DF (degree of freedom), Significant (*P* < 0.05), Non-significant (*P* > 0.05)

### Cellulase application

#### Evaluation of the detergent ability of cellulase

The detergent ability of the produced cellulase enzyme was carried according to Kumari et al. [29] with minor modifications. Consequently, the cellulase activity was estimated using scoured and the bleached cotton fabric was obtained from Weaving and Dyeing Co. El-Mehalla El-Kobora, Egypt as the substrate. The cotton fabric was cut into pieces (3 × 3 cm) and washed in 5 mL of cellulase enzyme with different concentrations of 0, 5, 10, 20, and 40 U/mL under the stirring condition at 1500 rpm for 1 h at room temperature. The detergent ability effect was evaluated using whiteness index % and SEM.

Color detection is an important factor that is used to evaluate the relevance of detergents [30]. The produce collected fabrics were washed with distilled water and dried in the oven at 70 °C overnight. The colorimetric analysis of the textile fibers was determined using a spectrophotometer with pulsed xenon lamps as a light source (Ultra Scan Pro, Hunter Lab, USA) 10˚ observers with D65 illuminant, d/2 viewing geometry, and a measurement area of 2.0 mm. The whiteness test was carried out to estimate the efficiency of the different concentrations of enzymes. Furthermore, the field emission-scanning electron microscope (FE-SEM) model (QUANTA FEG250, Netherlands) accelerated at high voltage (20 kV).

### Statistical analysis

The data in this study were represented as the means ± standard deviation (SD) and analyzed by one-way analysis of variance (ANOVA) using the statistical package SPSS, version 17.0.

## Results and discussion

### Demonstration of CMCase activity

**Fig. 1:**
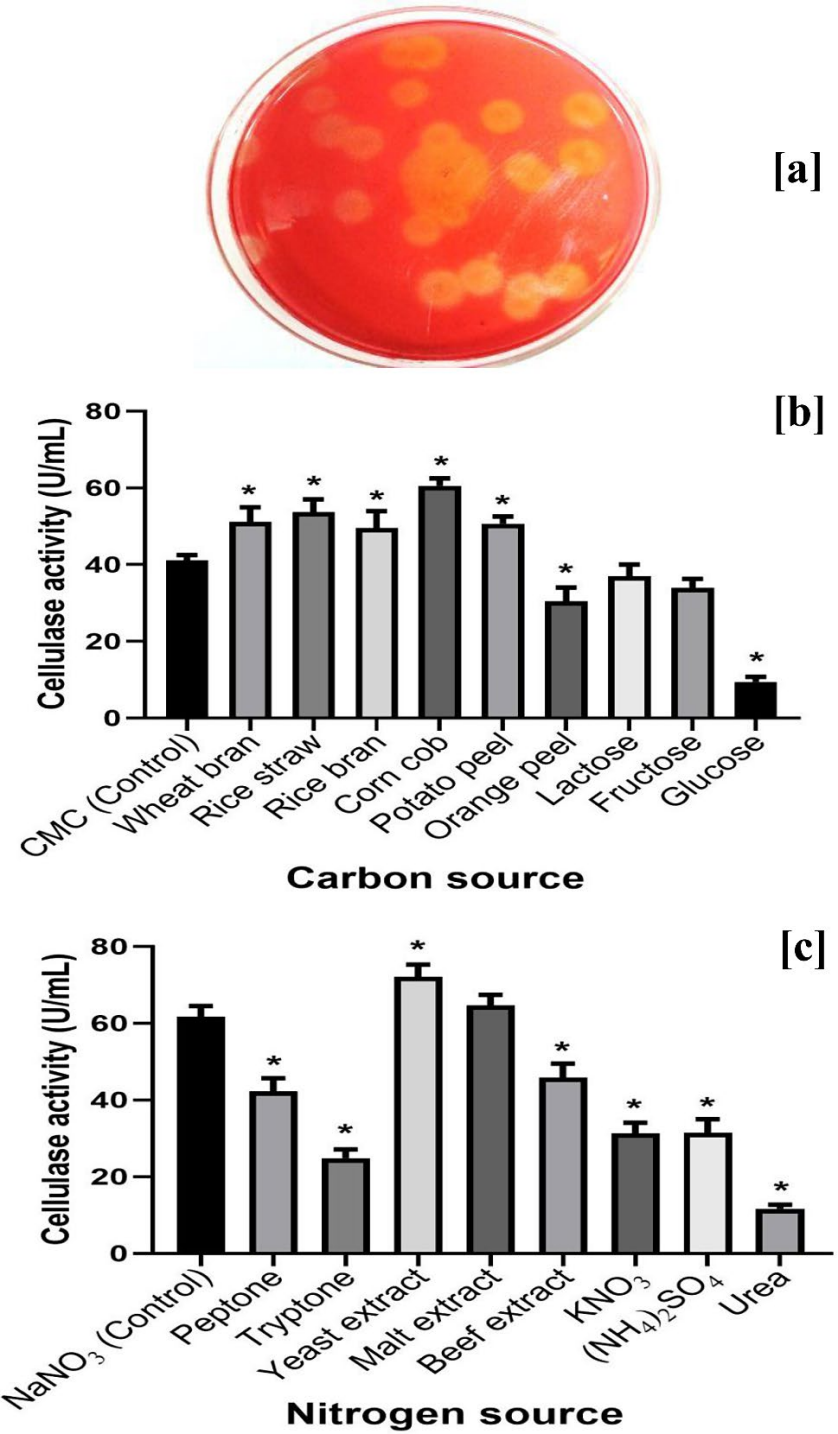
(**a**) Qualitative screening of *A. terreus* MN901491 on CMC-agar plate (**b**) Influence of various carbon sources (**c**) Influence of various nitrogen sources on cellulase productivity. The asterisk symbol (*) indicates the significant difference against the control at *P* < 0.05

The checking of cellulase production by *A. terreus* MN901491 was carried out after cultivation on CMC-agar plates stained with 1% CR solution. The results demonstrated that *A. terreus* MN901491 had the ability to produce cellulase by forming a halo zone about the fungal colonies after de-staining the plates with NaOH solution (1 N) due to CMC hydrolysis (Fig. 1a). Also, the cellulolytic activity was determined by growing the fungal strain in modified Czapek-Dox broth fermentation medium (basal medium) supplemented with CMC as a source of carbon that yield (47.2 U/mL) of cellulase enzyme.

### Optimization of cellulase production by OFAT

OFAT was applied to investigate various variables that may stimulate or inhibit enzyme production. This method depends on altering One- factor- at a time without testing the interaction between variables.

### Influence of various carbon sources on cellulase production

Diverse carbon sources involving synthetic and agricultural wastes were examined for superior yield of cellulase by *A. terreus* MN901491. As displayed in Fig. 1b, the maximum cellulase productivity (60.5 U/mL) was acquired by using corn cob followed by rice straw, wheat bran, potato peel and rice bran that yield (53.6, 51.1, 50.7 and 49.5 U/mL), respectively. According to the results, there was a significant increase in the yield of the enzyme by 1.47-fold when 1% CMC was substituted with 1% corn cob. On the other side, the lowest productivity of cellulase (9.4 U/mL) was observed after replacing 1% CMC with 1% glucose which significantly reduced the yield of the enzyme by 4.38-fold as presented in Fig. 1b. The utilization of agricultural wastes as carbon sources is considered a main goal to decrease the cost of industrial processes, solve the wastes accumulation problem, and reduce the environmental pollution [31]. Similarly, some agricultural residues like corn stover and wheat straw were found to give the highest cellulase production by *A. terreus* M11 and *A. flavus*, respectively [32, 33]. In contrast, Prasanna et al. [34] suggested that lactose was the most preferable carbon source utilized by *Penicillium* sp. for cellulase production.

### Influence of various nitrogen sources on cellulase production

Nitrogen source is represented as a critical factor for both growth and enzymatic synthesis in microorganisms [31]. The results illustrated in Fig. 1c showed that yeast extract was the superior source of nitrogen for enhancing cellulase productivity (72.4 U/mL) from *A. terreus* MN901491 followed by malt extract, beef extract, and peptone that produced (57.3, 45.8, 42.2 U/mL), respectively. The substitution of 0.2% NaNO_3_ with the same amount of yeast extract led to a significant increase in enzyme production by 1.2-fold. Otherwise, replacing 0.2% NaNO_3_ with the same amount of urea significantly decreased the productivity of cellulase (11.6 U/mL) by 82.2% as shown in Fig. 1c. In the present study it was noticed that, organic nitrogen sources were preferable for cellulase productivity by *A. terreus* MN901491 compared to the inorganic ones. Our results agreed with Alnusaire and Farag [24] who reported that, most organic nitrogen sources (particularly yeast extract and peptone) enhanced cellulase production by *A. ochraceus*. Also, yeast extract was the best nitrogen source for cellulolytic enzyme secretion by *Penicillium sp.* according to Prasanna et al. [34]. Other workers found that, when *P. oxalicum* R4 was grown in a culture containing ammonium sulfate, there was a high production of cellulase [35].

### Statistical designs for optimizing cellulase production

#### Plackett-Burman design (PBD)

PBD as introduced in Table 1 was carried out to screen and evaluate the major significant variables for cellulase productivity by *A. terreus* MN901491 [27]. The PBD matrix contains twelve runs with diverse levels of independent variables and the actual cellulase activity is displayed in Table 2. The results indicated that the greatest cellulase productivity (264.9 U/mL) was achieved in run 3 which is better than that gained from basal medium by 5.6-fold. The PBD was explained using the following first-order equation:

3 $$\eqalign{{\rm{Y = 98}}{\rm{.55}}\, - \,{\rm{21}}{\rm{.22A}} & \cr & {\rm{ + }}\,{\rm{15}}{\rm{.60}}\,{\rm{B}}\,{\rm{ + }}\,{\rm{54}}{\rm{.00}}\,{\rm{D}} \cr & - \,{\rm{18}}{\rm{.03}}\,{\rm{E}}\, - \,{\rm{9}}{\rm{.18}}\,{\rm{F}}\,{\rm{ + }}\,{\rm{31}}{\rm{.20}}\,{\rm{G}} \cr & {\rm{ + }}\,{\rm{33}}{\rm{.90H}}\, - \,{\rm{13}}{\rm{.22K}}\, - {\rm{7}}{\rm{.88}}\,{\rm{L}} \cr}$$

Where: Y, cellulase activity (U/mL); A, corn cob; B, rice straw; D, malt extract; E, K_2_HPO_4_; F, MgSO_4_.7H_2_O; G, KCl; H, (NH_4_)_2_SO_4_; K, incubation time; L, inoculum size.

The impact of the PBD was shown by ANOVA of cellulase productivity by *A. terreus* MN901491 as displayed in Table 3. ANOVA data involving *F*-value and *P*-value (probability value) was applied to prove the importance of statistical design and equation terms. The model *F*- value (103.19) refers to the significance of PBD. There is only a 0.96% chance that such a large *F*-value of the model could occur due to noise. On the other side, *P*-value < 0.05 implies that the regression model and its terms are significant. Depending on ANOVA results, corn cob, rice straw, malt extract, K_2_HPO_4_, KCl, (NH_4_)_2_SO_4_, and incubation time were considered effective (significant) variables that affect cellulase productivity by *A. terreus* MN901491. Moreover, the adequacy of the first-order regression model was estimated by coefficient of determination (*R*^*2*^). In the present study, the *R*^*2*^-value was very high (0.9879) which means, the statistical model can interpret 98.79% of the total variations in cellulase production. The adequate agreement of the predicted *R*^*2*^-value (0.9226) with *R*^*2*^-value (0.9879) and Adjusted *R*^*2*^-value (0.9782) proves the large correlation between observed and predicted values. In addition, the coefficient of variation (CV) value of 9.21% points to the accuracy of the regression model [31, 36].

**Fig. 2:**
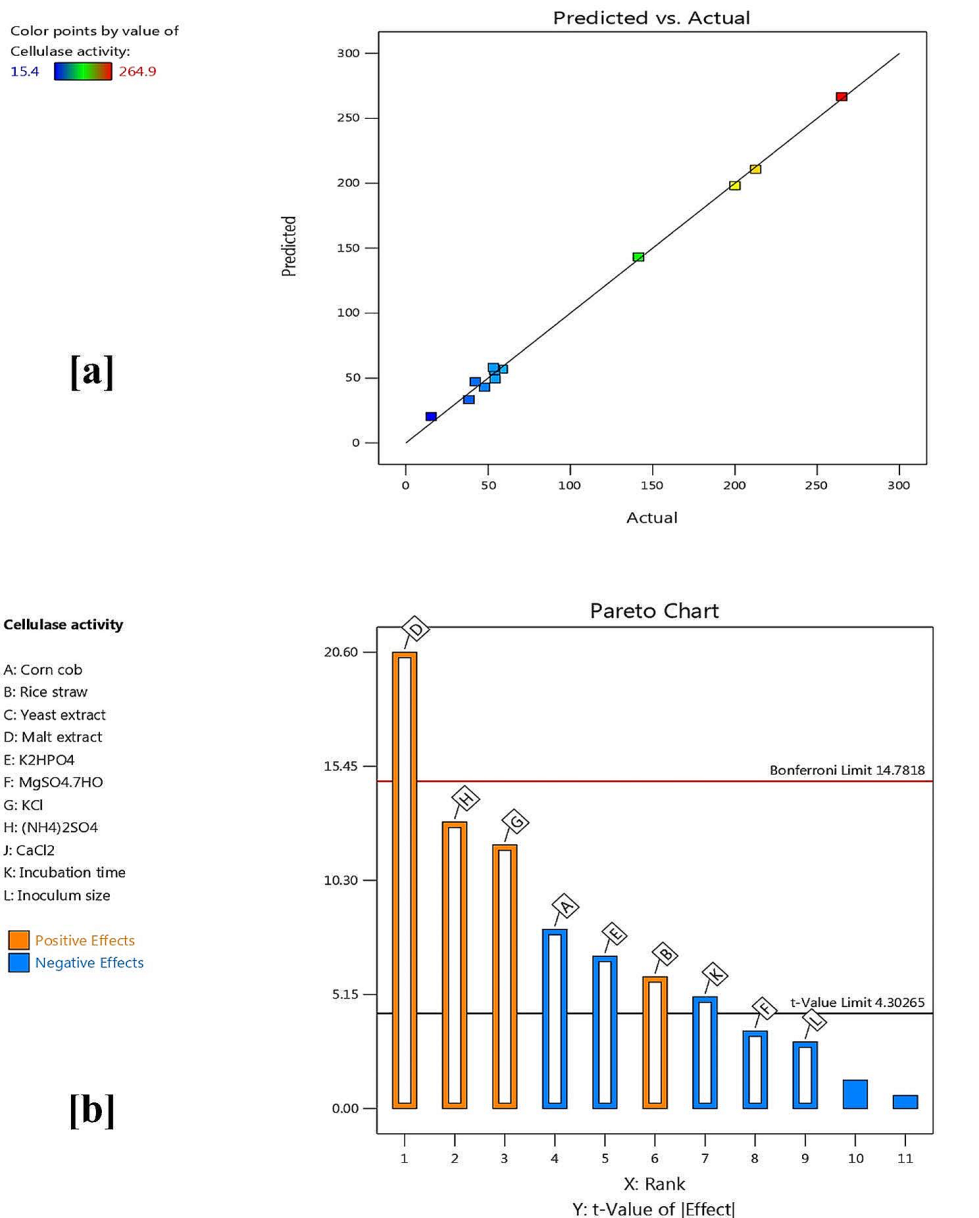
Relation among actual and predicted values of cellulase activity (**a**) Pareto Plot exhibits significant variables for cellulase production in PBD (**b**)

Furthermore, the predicted and actual response values were in consent and very closed as displayed in Fig. 2a which confirms the efficiency of the design model. The Pareto plot of PBD displayed the variables that possess a considerable significant effect in descending arrangement. As presented in Fig. 2b, the Pareto Plot indicated that malt extract, (NH_4_)_2_SO_4_, KCl and rice straw were the most significant variables which exhibited a positive impact on cellulase productivity whereas, corn cob, K_2_HPO_4_, and incubation time showed a negative effect. These data proved the deduced results of OFAT which revealed the ability of the fungal strain to utilize agricultural wastes (rice straw besides corn cob) as carbon sources for enhancing enzyme production. Also, the need for malt extract (nitrogen source) and other mineral salts that are fundamental for microbial growth and enzyme synthesis, was clear from the statistical analysis. Similarly, Alnusaire and Farag [24] reported that KCl has a positive effect while K_2_HPO_4_ has a negative influence on cellulase productivity by *A. ochraceus*. Also, our results are in accordance with Nisar et al. [12] who suggested that (NH_4_)_2_SO_4_ has a positively significant impact on cellulase production by *T. dupontii* TK-19 using SmF. In contrast, Nour et al. [37] reported that MgSO_4_, yeast extract, and CaCl_2_ have a considerable effect on cellulase production by *A. terreus* under SmF. On the other side, the variables including corn stover, wheat bran, (NH_4_)_2_SO_4_, KH_2_PO_4_ and MgSO_4_.7H_2_O had a non-significant effect on cellulase productivity from *A. niger* HQ-1 as offered by Zhang et al. [25].

#### Box-Behnken Design (BBD)

BBD was implemented to obtain the best concentration of significant variables [malt extract, (NH_4_)_2_SO_4_ and KCl] investigated by the PBD model and the levels (-, 0, +) of these variables were introduced in Table 4. According to the BBD, 15 runs were done and the experimental results of the examined variables were displayed in Table 5. The greatest cellulase productivity by *A. terreus* MN901491 was noticed in run 7 which gave 442.1 U/mL. A multiplied-statistical analysis of the experimental responses was carried out and the second-order model was interpreted as follows:

4 $$\eqalign{{\rm{Y}}\,{\rm{ = }}\,{\rm{405}}{\rm{.67}} & \cr & {\rm{ + }}\,{\rm{75}}{\rm{.47}}\,{\rm{A}}\,{\rm{ + }}\,{\rm{12}}{\rm{.75}}\,{\rm{B}} \cr & - \,{\rm{11}}{\rm{.10}}\,{\rm{C}}\, - \,{\rm{64}}{\rm{.25}}\,{\rm{AB}} \cr & - \,{\rm{3}}{\rm{.05}}\,{\rm{AC}}\, - \,{\rm{2}}{\rm{.30}}\,{\rm{BC}} \cr & - \,{\rm{104}}{\rm{.96}}\,{{\rm{A}}^2} \cr & {\rm{ + }}\,{\rm{28}}{\rm{.7}}\,{\rm{4}}{{\rm{B}}^2}\, - \,{\rm{46}}{\rm{.86}}{{\rm{C}}^2} \cr}$$

Where: Y, response (cellulase activity U/mL); A, malt extract; B, (NH_4_)_2_SO_4_; C, KCl.

**Fig. 3:**
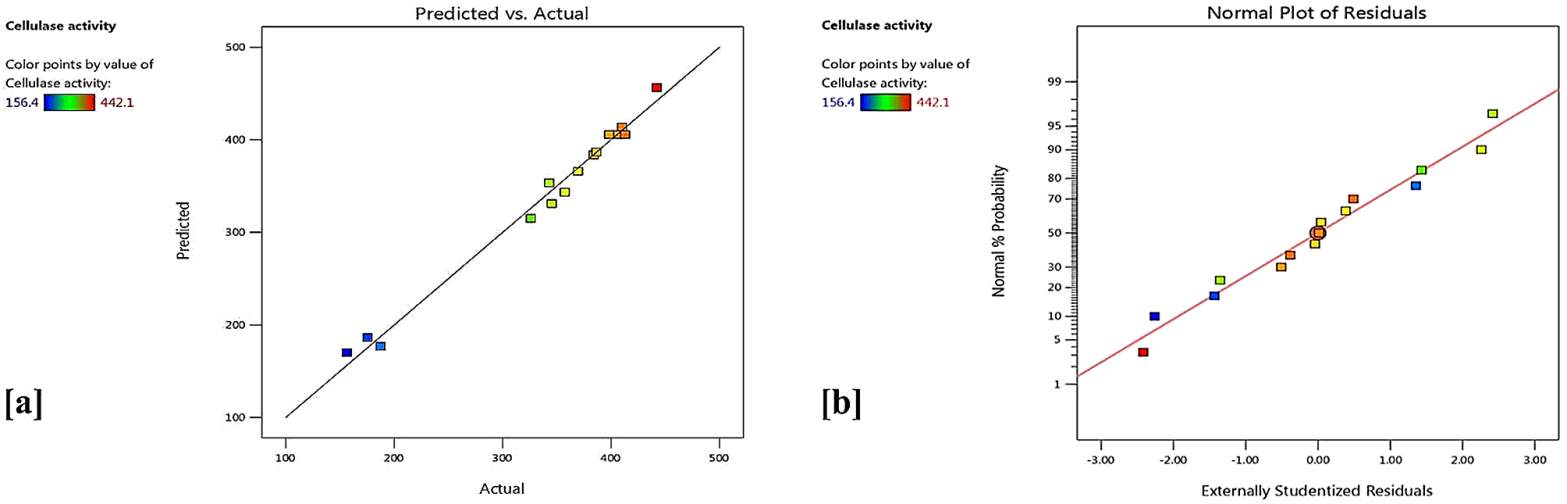
Relation among predicted and actual values of cellulase activity (**a**) Normal probability plot of the studentized residuals in BBD (**b**)

The ANOVA for BBD was applied to achieve the importance of the regression model and equation terms (variables). As seen in Table 6, the lower probability value of the model (*P*-value < 0.05) and its *F*-value of 46.82 indicate the model was significant. There is only a 0.03% chance that such a large *F*-value of the model could occur due to noise. According to the results, A, AB, A^2^, B^2^, and C^2^ were represented significant model terms. Furthermore, the *R*^*2*^-value (determination coefficient) can demonstrate the adequacy of the regression model and the variability of the results. So, the model with *R*^*2*^-value > 0.9 indicates that there is a great correlation between the actual and predicted responses [31]. The *R*^*2*^-value of (0.9883) implies the model can explain 98.83% of the whole differences in cellulase productivity. Also, the Adjusted *R*^*2*^-value (0.9672), predicted *R*^*2*^-value (0.8250) and CV value (4.89%) confirmed the efficiency of the regression model and provided a good interpretation of the experimental results. On the other hand, the lack of fit was non-significant which means the statistical model had appropriate fitness and it was significant. Moreover, the model validation was checked through closing among experimental and predicted values of cellulase activity as presented in Fig. 3a. Also, the normal probability plot of the residuals displayed the proximity of the plotted points from a straight line that proves the model suitability to the experiments (Fig. 3b).

**Fig. 4:**
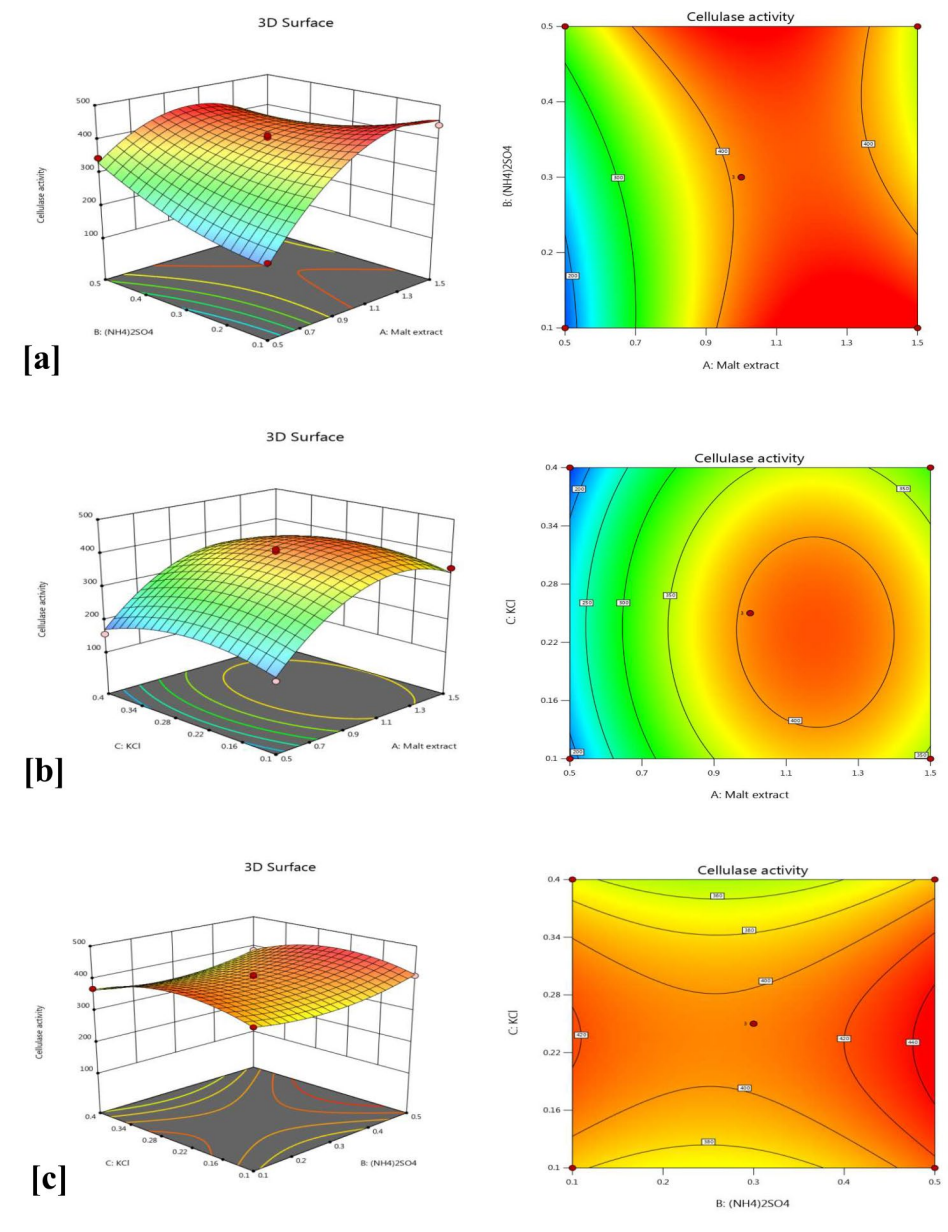
Response surface 3D and contour plots showing interaction between each two variables influencing cellulase productivity by *A*. *terreus* MN901491 (**a**) Malt extract and (NH_4_)_2_SO_4_ (**b**) Malt extract and KCl (**c**) (NH_4_)_2_SO_4_ and KCl

The interaction between independent variables and their actions on cellulase productivity was investigated by contour and three-dimensional (3D) surface plots. In these charts, enzyme activity was drawn on the z-axis against two variables, while the other variable was maintained at the central value. Figure 4a exhibits the action of malt extract and (NH_4_)_2_SO_4_ on cellulase production by *A. terreus* MN901491, whereas KCl was preserved at its central value (0.25%). In this case, the maximum cellulase activity (442.1 U/mL) occurs at a high level (1.5%) of malt extract and a low level (0.1%) of (NH_4_)_2_SO_4_. Also, Fig. 4b displays the influence of malt extract and KCl on cellulase productivity, whereas (NH_4_)_2_SO_4_ was kept at its central value (0.3%). The highest cellulase activity (405.9 U/mL) was obtained at central levels of both malt extract (1%) and KCl (0.25%). Furthermore, Fig. 4c shows the interaction between (NH_4_)_2_SO_4_ and KCl keeping malt extract at its central level (1%). The maximum cellulase activity (410.2 U/mL) takes place at a high level (0.5%) of (NH_4_)_2_SO_4_ and a low level (0.1%) of KCl.

The final medium composition after optimizing cellulase productivity by *A. terreus* MN901491 was (g/L): corn cob, 5; rice straw, 5, yeast extract, 2; malt extract, 15; (NH_4_)_2_SO_4_, 3; MgSO_4_.7H_2_O, 0.5; K_2_HPO_4_,0.5; KCl, 2.5 at 30 °C, pH 6, agitation speed 150 rpm and incubation time 5 days. In the present work, the maximum cellulase productivity (442.1 U/mL) by *A. terreus* MN901491 using BBD was 9.3-fold greater than that obtained from the basal medium. From the previous results, the optimization process including OFAT and statistical design methods played an important role in the improvement of cellulase yield from *A. terreus* MN901491. Our result is higher than that mentioned by Alnusaire and Farag [24] who suggested that optimized medium promotes cellulase production from *A. ochraceus* by 2.87-fold. Also, cellulase production by *A. niger* HQ-1 increased by 2.5-fold after optimizing medium parameters [25]. Otherwise, our result is lower than Sorour et al. [38] who acquired 22.7-fold in cellulase productivity by *A. penicillioides* 12 ASZ using statistical designs.

### Assessing cellulase in detergent ability

The bio additive detergent usually included in the process involves bio-based or bio-origin detergent such as microbial enzymes. Indeed, the bio-additives to detergent agents play an important role in worldwide health that has good compatibility with skin and prevent dermatitis in cases hypersensitive to synthetic detergents [39]. Besides, biological additives such as enzymes to detergent agents offer nice environmental benefits where these materials are completely ecofriendly [40, 41].

**Fig. 5:**
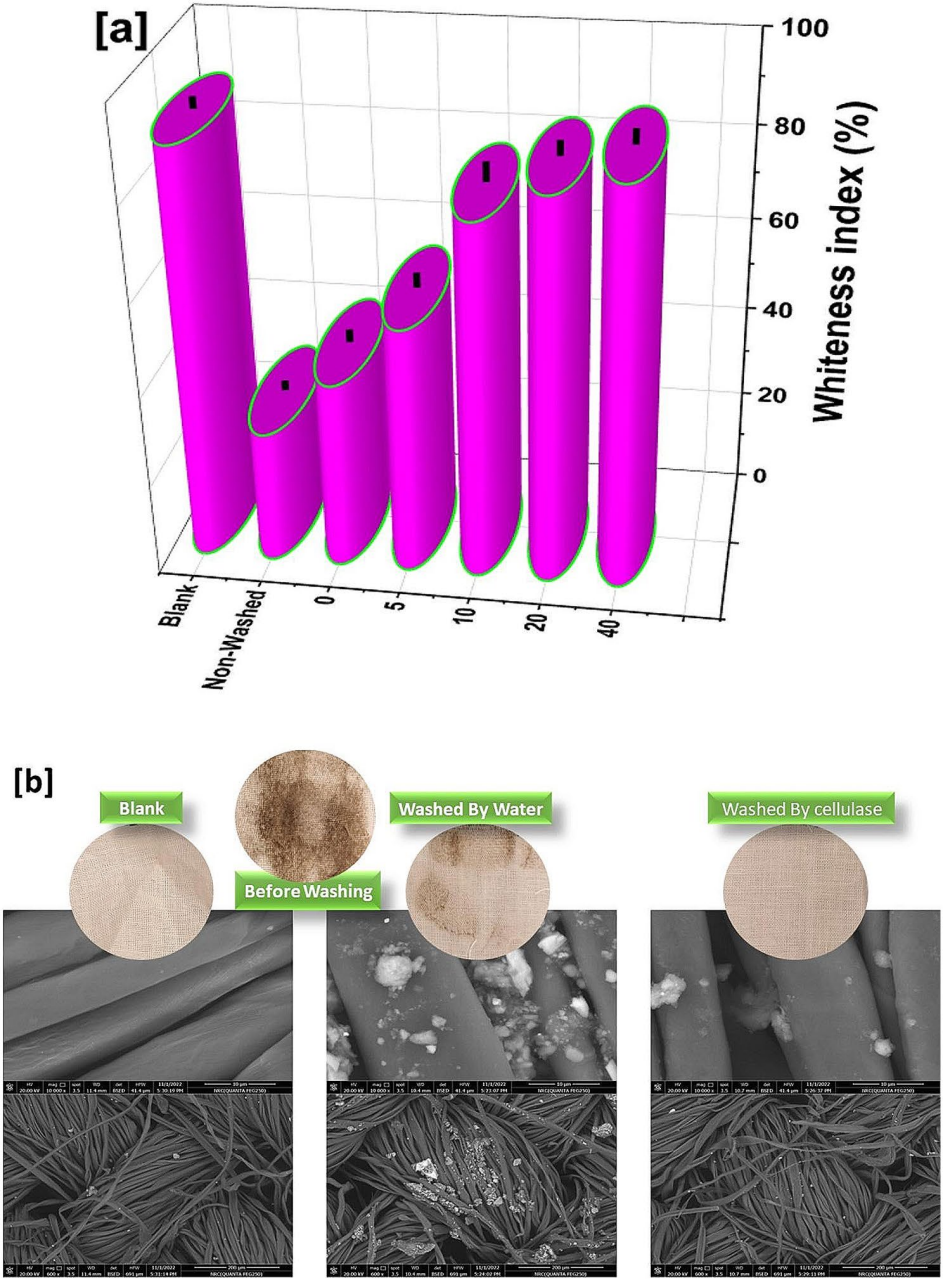
The whiteness index of the blank, non-washed and washed cotton fabric with different cellulase concentrations (**a**) The SEM images of blank and washed cotton fabric with 0 and 20 U/mL of cellulase enzyme (**b**)

The detergent ability of cellulase enzyme is acting via the cutting efficiency of the cellulose fibers tiny terminals that are attached to undesirable particles [19]. In this context, the whiteness index of the blank, non-washed and washed cotton fabric with different cellulase concentrations (concentration zero is referred to water only) was studied using the whiteness index as well as SEM as presented in Fig. 5. The whiteness of treated and blank cotton fabric was shown in Fig. 5a and blank high whiteness index value of nearly 90% as well as the water washed sample reflected whiteness about 40% that closely to non-washed cotton fabric [42]. Otherwise, the non-washed cotton fabric recorded the lowest value of the whiteness index which was less than 30%. In this context, the treated cotton fabric via cellulase enzyme observed a significant difference according to the treated concentrations. The high concentrations ranging from 10 to 40 U/mL conferred a high detergent activity in comparison with 5 U/mL enzyme concentrations. These observations emphasized the efficiency of the cellulase enzymes as a detergent agent for cellulosic fibers with optimum concentration at 20 U/mL [43].

On the other hand, the SEM study was presented as images with low and high magnifications for blank and washed cotton fabric with 0 and 20 U/mL of cellulase enzyme as seen in Fig. 5b. The blank cotton fabric was observed as a typical appearance in comparison with other images in the literature [44, 45]. Moreover, the washed sample with water (zero enzyme concentration) observed a cluster of particles over fibers that are distributed randomly in between and over the fibers. On the other side, the washed cotton fabric with 20 U/mL confirmed a good appearance of fibers that is clean without aggregations of particles over fibers. Indeed, these observations affirmed that the efficiency of the cellulase enzyme that eliminated the undesirable particles over the fibers enhanced the fiber’s whiteness.

## Conclusion

The presented work was stabilized as a multi-efficiency in which agricultural wastes were used to produce cellulase enzyme from fungal strain, *Aspergillus terreus* MN901491 for bio-additive detergent applications that involved ecofriendly and green processes. Also, the parameters affecting cellulase productivity were optimized using OFAT and statistical designs (PBD and BBD) methods. The final optimized medium enhanced cellulase productivity from *A. terreus* MN901491 by 9.3-fold greater than that gained by the original medium. Further, the efficiency of cellulase activity as detergent additives was evaluated and estimated using whiteness and scanning electron microscope (SEM) which exhibited a nice performance in both the morphological and physical appearance of the cotton fabrics. The future perspective of such kind of detergent additives can be followed by using different types of enzymes with various conditions to be close to being used in industrial applications.

## Data Availability

No datasets were generated or analysed during the current study.
